# A Brief Reminder of Systems of Production and Chromatography-Based Recovery of Recombinant Protein Biopharmaceuticals

**DOI:** 10.1155/2019/4216060

**Published:** 2019-01-08

**Authors:** B. Owczarek, A. Gerszberg, K. Hnatuszko-Konka

**Affiliations:** Department of Molecular Biotechnology and Genetics, Faculty of Biology and Environmental Protection, University of Lodz, Banacha 12/16, 90-237 Lodz, Poland

## Abstract

Recombinant proteins are produced for various applications in laboratory and industrial settings. Among them, therapeutic applications have evolved into a mature field in recent years, affecting the face of contemporary medical treatment. This, in turn, has stimulated an ever-greater need for innovative technologies for the description, expression, and purification of recombinant protein biopharmaceuticals. Therefore, many biopharmaceuticals are synthesized in heterologous systems to obtain satisfactory yields that cannot be provided by natural sources. As more than 35 years has passed since the first recombinant biopharmaceutical (human insulin) successfully completed clinical trials in humans, we provide a brief review of the available prokaryotic and eukaryotic expression systems, listing the advantages and disadvantages of their use. Some examples of therapeutic proteins expressed in heterologous hosts are also provided. Moreover, technologies for the universal extraction of protein molecules are mentioned here, as is the methodology of their purification.

## 1. Introduction

Human cells produce an enormous number of proteins, and dysfunction in these may lead to serious diseases and developmental abnormalities. To treat these protein deficiencies the missing or dysfunctional molecules are complemented or substituted with therapeutics provided by different biological systems. However, protein therapeutics must unavoidably adhere to quality constraints that are much stricter than those for chemical industries [1, 2]. Although it is undoubtedly a challenging task to obtain an active protein in a way that is economically feasible, biopharmaceuticals (recombinant proteins, monoclonal antibodies, or vaccines) are the largest group of drugs developed in the pharmaceutical industry [3]. Market calculations estimated the recombinant protein drug industry to be around 10% of the entire drug market, predicting an even larger proportion in the future [4]. The global market of biopharmaceuticals is estimated to grow at a Compound Annual Growth Rate (CAGR) of 13.8% from 2018 to 2025, according to the latest report developed by Allied Market Research. The report “Biopharmaceuticals Market by Type and Application: Global Opportunity Analysis and Industry Forecast, 2018-2025” presenting an analysis of profiles of the major players (e.g., Biogen, Inc., Johnson & Johnson, Novo Nordisk A/S, or Pfizer, Inc.) projects that the global biopharmaceuticals market, which reached $186,470 million in 2017, will have reached $526,008 million by 2025. The more detailed characteristics of the expected value growth of particular biologic types in the biopharmaceuticals market (divided into dominating monoclonal antibody, growth and coagulation factor, interferon, vaccine, insulin, erythropoietin, and hormone) are available in the report and on the Allied Market Research website [5].

The technologies behind the synthesis of biopharmaceuticals have changed since several protein drugs were approved in the 1980s, although protein molecules have been used as biopharmaceuticals since the 1920s (reports on insulin from pig pancreas) [6] and there were several milestones in the utilization and development of various expression systems. In 1982, the date when Humulin (human insulin) was approved by the Food and Drug Administration (FDA) as the first recombinant biopharmaceutical was recognized as a scientific turning point in this review. That was the point at which genetic and biotechnological research finally met medical needs and standards, consequently providing a new generation of therapeutics. Therefore, after more than 35 years, we present a brief overview of the existing expression platforms, together with the main extraction and purification methods.

It should be noted here that the terminology connected to biopharmaceuticals varies between scientific communities or industrial units, sometimes referring to different subcategories of therapeutics within the general category. In the context of this review the term “recombinant protein biopharmaceuticals” includes any pharmaceutical protein drug (e.g., recombinant proteins and peptides, vaccines, and monoclonal antibodies) that was obtained via engineering of biological sources.

## 2. Comparison among the Production Systems of Recombinant Protein Therapeutics

A wide range of platforms is available for production of recombinant proteins and each of the systems offers many amenities. Recombinant biopharmaceuticals can be synthetized in prokaryotic and eukaryotic cells including bacteria, yeasts, insect cells, filamentous fungi, microalgae, mammalian cells, and transgenic animal and plant organisms [7, 8]. The properties of the target protein—its structure and biological activity—dictate the choice of its production platform. The better a protein of interest is characterized, the easier it can be extracted and purified.

The main difference between possible various expression systems reflects the anatomy of a cell. Bacterial cells do not possess a nucleus, endoplasmic reticulum (ER), or Golgi apparatus, which play a substantial role in transport and posttranslational modification (PTM). The humanlike splicing of mRNA is usually provided by mammalian or insect cells. However, working on these hosts might be too time-consuming and expensive. Hence, other systems may turn out to be more affordable and efficient. Therefore, great attention should be paid to the choice of platform for the production of recombinant pharmaceuticals to gain scalability and high yield. In this review, systems for the production of recombinant proteins are concisely characterized and compared. A comparison of the production systems is presented in Table 1.

### 2.1. Bacteria

The bacterial system of the production of recombinant protein therapeutics is believed to be attractive on account of low costs, rapid bacteria growth, and medium productivity.* Escherichia coli* is a model organism characterized with well-known biochemistry and genetics; hence it is no surprise that this host is predominant among the bacterial platforms of expression (e.g.,* Pseudomonas *or* Streptomyces *system). Genetic manipulations in* E. coli* are simple and straightforward. When the production of recombinant therapeutics concerns membrane proteins, the* Lactococcus lactis* system is commonly considered [9, 10]. The bacterial system is easy to culture and low priced. The first engineered biopharmaceutical for diabetes treatment, human insulin (Humulin, approved by the Food and Drug Administration (FDA) in 1982), appeared in the early 80s and was produced in* E. coli* cells [3]. Today, interferons, a human growth hormone, a tumor necrosis factor, and interleukins are among the proteins produced in the* E. coli* system [11, 12]. Small cytosolic proteins and polypeptides coded by less than a hundred nucleotides are expressed as fusion proteins to obtain the highest efficiency [10]. Using* E. coli* for the production of protein therapeutics usually allows the optimization step to be skipped, since many standard plasmid vectors can be adopted easily.* E. coli* and bacteriophage T7 RNA polymerase is a commonly used expression system, which is even recommended as a “*what-to-try-first*” approach by some of the structural genomics consortia. The system was recommended to express globular, soluble eukaryotic, and prokaryotic proteins since it significantly enhances the level of protein expression [10].

Unlike proteins produced in eukaryotic cells, molecules synthetized in* E. coli* do not undergo posttranslational modifications (PTMs) such as glycosylation, formation of disulfide bonds, phosphorylation, or proteolytic processing (although disulfide bonds could be formed using the plasmid that sends a target protein to the periplasmic membrane). Since PMTs are involved in folding processes, stability, and biological activity, a bacteria-derived recombinant protein might be inactive. However, attaching synthetic PTSs might be the solution to this problem [11]. Another problem concerning the system of bacterial expression is posed by genes with rare codons, which can be found, for example, in the human genome but are uncommon in that of bacteria. Their expression is often low and triggers premature termination of the synthesis of a protein molecule. It can be solved by rare codon site-directed replacement (or using* E. coli* plasmid for rare codons as well) [11]. Finally, bacteria overproducing recombinant proteins are susceptible to conformational or metabolic stresses, which affects protein solubility negatively. To transfer a protein molecule into a soluble form one should take the following factors into account: appropriate temperature, medium composition, number of plasmid copies, or strength of the promoter used [11].

### 2.2. Yeasts

Scientists seeking a highly effective system of recombinant therapeutics production also tried simple eukaryotic organisms: yeast.* Schizosaccharomyces pombe*,* Kluyveromyces lactis*, or* Yarrowia lipolytica *are used for this purpose, although* Saccharomyces cerevisiae* is the most popular among yeast expression vectors [10]. Both bacteria and yeasts are relatively economically feasible, fast-growing systems, which can be cultured in bioreactors with high cell density [11]. Human serum albumin, insulin and its analogs, and vaccines against papillomavirus and hepatitis are the main biopharmaceuticals obtained in yeasts [13].

The yeast platform is commonly used when a therapeutic protein is synthetized in an insoluble form in bacteria and glycolytic promoters like pTDH3 or galactose-induced promoters (e.g., pGAL1) are typically used [13]. The fact of being eukaryotes gives them an advantage in the case of posttranslational modifications. Hence, yeasts perform O-linked phosphorylation, glycosylation, acetylation, and disulfide bond formation, for instance. However, they are unable to provide high-mannose type N-glycosylation as in the case of cells of higher eukaryotes. Using genetic engineering, yeasts were programmed to carry out humanlike N-glycosylation, proving that this platform is very promising and worth developing [11, 13]. Furthermore, the production of recombinant protein therapeutics in yeasts facilitates the purification step, since the pharmaceutical can be secreted to the medium [13]. On the other hand, yeasts, like bacterial systems, are subjected to conformational stress, leading to defective conformation of the final product.

### 2.3. Microalgae

Microalgae represent another platform for the production of recombinant proteins. The most attention is focused on* Chlamydomonas reinhardtii,* although there are reports regarding other microalgal species, including* Phaeodactylum tricornutum*, the red alga* Cyanidioschyzon merolae*, and the green algae* Haematococcus pluvialis* and* Dunaliella tertiolecta* [14].* C. reinhardtii* is an extremely attractive alternative due to being low cost as well as low tech. A very efficient technology to produce biomass from these unicellular photosynthetic algae has been recently developed using photobioreactors [15]. Furthermore, breeding algae in closed bioreactors under controlled conditions allows the desired biomass to be obtained and avoids the risk of contamination [14]. It is not only the possibility of obtaining large biomass in a short time that makes them an attractive host for the production of a wide range of biopharmaceuticals, but also many other features characteristic of these single-cell algae.* C. reinhardtii* is a model organism whose three genomes (nuclear, mitochondrial, and chloroplast) have been sequenced, giving rise to its targeted genetic modification [15]. Undoubtedly, it should be emphasized that, in the case of algae, the expression of a transgene can be obtained in both the nuclear and chloroplast genome, which makes this organism a universal host. However, introduction of a gene of interest (GOI) into the small chloroplast genome is much more desired, because it ensures stable and high-level expression [14], while in other microalgae organelles such glycosylation could occur a little differently compared to that in mammalian cells [16]. Thus, the chloroplast genome can serve as a safe subcellular compartment for the accumulation of the recombinant protein at a high level. Moreover, this unicellular alga offers additional advantages, including proper folding as well as disulfide bond formation, which is crucial for correct protein assembly [15]. Due to all these features, algae are used for the production of complex recombinant proteins, as well as edible vaccines, which significantly reduces production costs [15]. With the use of genetic engineering tools, more than 100 recombinant proteins including monoclonal antibodies (mAbs), subunit vaccines, growth factors (e.g., hGH), immunotoxins, and antibody mimics are produced in* C. reinhardtii* [14, 16].

### 2.4. Filamentous Fungi

Filamentous fungi serve as a cell platform mainly for the production of large-scale industrial enzymes. However, due to certain features, such as very fast and strong growth and the ability to release large amounts of proteins directly to the substrate, this platform is used more widely for the production of recombinant proteins [17, 18]. It was reported that fungi such as* Aspergillus niger* were able to produce and secrete c.a. 30 g/L of glucoamylase and* Trichoderma reesei* even 100 g/L of extracellular proteins [17]. However, some disadvantages of using filamentous fungi as a host platform for recombinant proteins production have been suggested, including too low a level of transformation frequencies, changes, or morphological defects. Furthermore, the final product may differ from the mammalian protein, due to the impact of incorrect pH, fungal proteases activity, or differences in the glycosylation pattern [17, 19]. Regarding glycosylation problems, some successful attempts were made in the case of* A. nidulans* and* A. niger* [19]. Similarly, in the case of* Neurospora crassa*, in order to avoid proteolytic degradation of the final product, the entire production process was optimized, including crucial parameters such as pH which affects the activity of fungal proteases [17]. Currently,* A. nidulans*,* A. niger*,* N. crassa*, and* T. reesei* are used mainly as expression systems for obtaining recombinant proteins (e.g., antibodies) [17, 18, 20].

### 2.5. Insect Cells

An insect cell (IC) platform appears to be a compromise solution between two other systems: bacterial or mammalian cells. The production of recombinant proteins in insect cells is possible thanks to the development of the baculovirus expression vector system (BEVS) [7]. The process involves two stages. First, the insect cells are multiplied to the desired concentration and then infected with a properly modified baculovirus containing GOI [21]. The insect cells used in this system originate from* Spodoptera frugiperda*,* Drosophila melanogaster*, and* Autographa californica* and they are susceptible to infection by baculovirus [21, 22]. Other less-used insects are* Trichoplusia ni* or* Bombyx mori* [22]. Different therapeutic proteins, such as tissue plasminogen activator (tPA), human glutamic acid decarboxylase (hGAD65), and viral and parasitic proteins, were obtained with the use of BEVS-IC [21, 22].

The IC system has many advantages as far as the expression of foreign proteins is concerned. Since the insect cell lines used for recombinant protein production grow fast to obtain high density in comparison to mammalian cells, a smaller volume of their cultures is necessary [22]. Moreover, in the case of insect cell lines, there is no risk of contamination by prions and oncogenic DNA [23] and they ensure a high yield of proteins [21]. In insect cells, as in mammalian ones, signal peptides are cleaved and disulfide bonds are formed in the endoplasmic reticulum [7]. However, if the desired protein requires complex posttranslational modifications, the insect cell system will not be an ideal solution. The main obstacle is the differences in the glycosylation pattern, which significantly affect the biological activity of the protein [23]. Insect cells are not able to carry out N-glycosylation [7, 23]. To overcome this obstacle, different approaches were tried, including introduction of mammalian glycosyltransferases into insect cells or coexpression of these enzymes together with GOI in baculoviruses [23]. Despite many benefits, this system is rather expensive due to the costs generated by the media for culturing insect cells [21].

### 2.6. Mammalian Cell Culture

In the case of clinical applications the expression of recombinant proteins in mammalian cells dominates the other systems [10]. Mammalian host cells are the source of enzymes, monoclonal antibodies, clotting factors, hormones, and cytokines [24]. Their production necessitates an appropriate cell line and a methodology of transporting the gene of interest into the cells. Usually, a foreign DNA is introduced into an animal cell using one of two main methods. The first involves a virus infection. The second method does not use any vectors and the heterologous DNA is introduced directly into the cell with the use of, e.g., microinjection and electroporation techniques. Promoters from* Cytomegalovirus* and the simian virus are widely used for constructing the expression cassettes for mammalian cells [10].

Mammalian cell cultures are capable of synthesizing large and complex protein molecules. The most commonly used lines of cell cultures are mouse myeloma and Chinese hamster ovary cell systems. However, there has been a shift to human cell lines recently (although HeLa, the first human cell line, was developed in 1951). It increases the probability that the target protein will gain PTMs characteristic of human proteins. It should be noted here that, in general, other mammalian lines could also perform appropriate posttranslational modifications, although nonhuman PTMs may be produced as well (for example, N-glycolylneuraminic acid or galactose-*α*-1,3-galactose, which can trigger the immune response) [24].

Another advantage of mammalian cultures consists in the possible secretion of heterologous proteins in the site of extraction via cell lysis. Moreover, the mammalian system for protein expression is characterized by a high tolerance to changes in temperature, oxygen, pH, or pressure level in the production stage [24].

However, the system is not free of limitations, namely, the risk of infection by animal viruses or the low level of production process [6, 24]. Finally, the medium dedicated to cell lines poses yet another difficulty. It was estimated that this kind of cell line required over fifty various components, making it hard to optimize their concentrations. Mammalian cells often require supplementation of growth factors, amino acids, reducing agents, or vitamins, while microbes frequently require just a simple combination of basic elements, such as nitrogen, carbon, phosphorus, or mineral salts [25], and tend to be more fast growing. According to the review by Wiktorek-Smagur et al. [1], the time needed to obtain a recombinant protein is short in the case of the bacterial system and long in the mammalian cell cultures with the yeast system situated in the middle [26].

### 2.7. Transgenic Animals

In 2006 the European Medicines Evaluation Agency approved the first recombinant protein biopharmaceutical secreted in the milk of transgenic goats: antithrombin (AT). Antithrombin prevents overactivity of the coagulation system [27]. Since that time transgenic animals have enabled the synthesis of a wide range of recombinant therapeutics, including monoclonal antibodies, vaccines, cytokines, hormones, enzymes, growth factors, fibrinogen, and collagen [6]. Nowadays, two main ways of sourcing proteins are in use: milk from transgenic mammals (such as sheep, goats, cows, rabbits, or pigs) and eggs from transgenic chickens. Additionally, there are other systems for production, e.g., the blood, urine, and silk gland, hemolymph of insect larvae, or seminal plasma [6].

The aim of the genetic engineering approach in this case is to obtain transgenic animals with a transgene coding a recombinant protein integrated into the genome of all their cells and capable of passing it on to their offspring. Usually, the DNA integration into the genome of the cell occurs in gene-poor regions. This can be obtained via microinjection of a transgene sequence into a male zygote pronucleus or by injecting the “somatic” nucleus into an oocyte devoid of its own nucleus [26, 27].

The production of therapeutics based on transgenic animal platforms has distinct advantages, such as natural secretion (like, e.g., in the case of milk) and providing of correct posttranslational modifications. Of course, the immune response can also be triggered when a PTM is not normally performed by a host. However, producing transgenic animals is ethically questionable. Some protein products may influence their health, as was shown in the case of the human growth hormone or erythropoietin (the coding transgene was expressed in rabbit milk) [6].

### 2.8. Transgenic Plants

The production of recombinant proteins harboring plant expression machinery started over 30 years ago when the human growth hormone was obtained in tobacco plants [28]. Nowadays, three main strategies are employed to produce recombinant proteins: cell cultures, plant tissue-based systems, and construction of transgenic plants. The transformation methodology includes using bacterial (agroinfection) or viral infection or direct approaches such as biolistic bombardment or the PEG-mediated technique [29].

Using plants for sourcing recombinant biopharmaceuticals has the potential to increase their production and decrease costs. Plant factories possess numerous advantages widely discussed in the literature: low cost, safety, high stability of engineered proteins, their insensitivity to minor fluctuations of pH or temperature, presence of metabolites, capability of producing N-glycosylated proteins, and easier and cheaper storage of engineered drugs [30]. Importantly, the overall cost of producing transgenic plants synthesizing recombinant protein is low, while a high yield is maintained. In comparison to prokaryotic and other eukaryotic systems, it might be 10 to 50 times lower [30, 31]. Plant leaves, fruit, or seeds are hypothetically unlimited sources of therapeutic proteins. The expression levels (defined as total soluble proteins (TSPs)) from engineered plants range from 0.001% to 46.1% [32]. The possibility of a pharmaceutical accumulation in a chosen cell compartment (mainly in the endoplasmic reticulum) or plant organ is a great advantage of the transgenic plant system not found in the others [1, 33]. Moreover, the risk of contamination (with animal pathogens) is reduced in the case of in planta expression. In contrast to prokaryotic cells, plant organisms are able to provide posttranslational modifications that, in turn, provide protein biological activity [1]. However, host-specific variances in glycan structures are distinguished between molecules matured in plants and humans. Human pharmaceutical proteins synthesized in plants often produce a plantlike rather than humanlike glycosylation pattern. To overcome this problem the strategy of glycoengineering plant expression systems is being developed [34].

On the other hand, the system must face some challenges, e.g., difficulties in controlling the transgene expression level (it may vary in plant organs, plant tissues, or subsequent generations). Furthermore, the purification stage is more complex since “content” such as secondary metabolites or pesticides must be removed. Hence, 80%–90% of the cost of producing engineered protein might be generated by downstream processing [31]. However, purifying and storing steps could be eliminated in the case of edible vaccines, where the food becomes a vaccine itself (the genetically modified plant starts producing pathogen components resulting in immunization of the consumer against a particular disease) [31, 35]. The list of candidate plants investigated as a potential source of edible vaccines includes rice, bananas, peas, potatoes, lettuce, and corn. At present, there are sixteen types of antigens against various diseases such as gastroenteritis, rotavirus, rabies, or cholera produced in planta [35]. Biopharmaceuticals produced in plants are at various stages of clinical trials or market implementation [31, 36–38]. Selected examples are presented in Table 2.

As mentioned earlier, exploiting plant platforms for producing high-value proteins has many advantages, including the possibility of easy scale-up. It should be emphasized that research work on new therapeutic preparations at an economically feasible level is a multistage process. It involves, among other things, estimating market demand, choosing the right expression system, and estimating production costs. Four basic stages of developing the technology of therapeutic protein production through molecular farming are distinguished: identification of the desired protein, introduction of the gene coding this protein, optimization of the expression of the gene encoding the desired protein (production on a laboratory scale), and protein production on an industrial scale (plants bioreactors) [39]. While the first step is not complicated, the remaining stages require meticulous elaboration of all details. In the case of plant platforms for the production of recombinant proteins, their too low content in plant tissues remains the main problem. Therefore, experimental work on improving the productivity of plant systems of numerous biopharmaceuticals is ongoing. This research involves selecting the appropriate plant system, as well as a suitable promoter, regulatory sequence, or signal sequence that sorts proteins into cell compartments or by means of secretion [40].

Currently, two methods are mainly used to introduce the structural genes encoding the desired therapeutic protein, namely,* Agrobacterium*-mediated transformation or via plant viral vectors [39]. In the first method, transgenic plants or plant cells with a stable transgene expression are obtained, while in the second case transient expression of the foreign gene is obtained. In both ways, the protein is extracted and purified and then examined in terms of its activity or immunogenicity in laboratory conditions on animals. Additionally, in the case of transgenic plants, the extraction step can be omitted, because some parts of plants could serve as an edible vaccine. Subsequently, in the case of the development of an efficient system for the production of a given therapeutic (fully functional) protein, clinical trials are carried out [31, 41].

**Figure 1:**
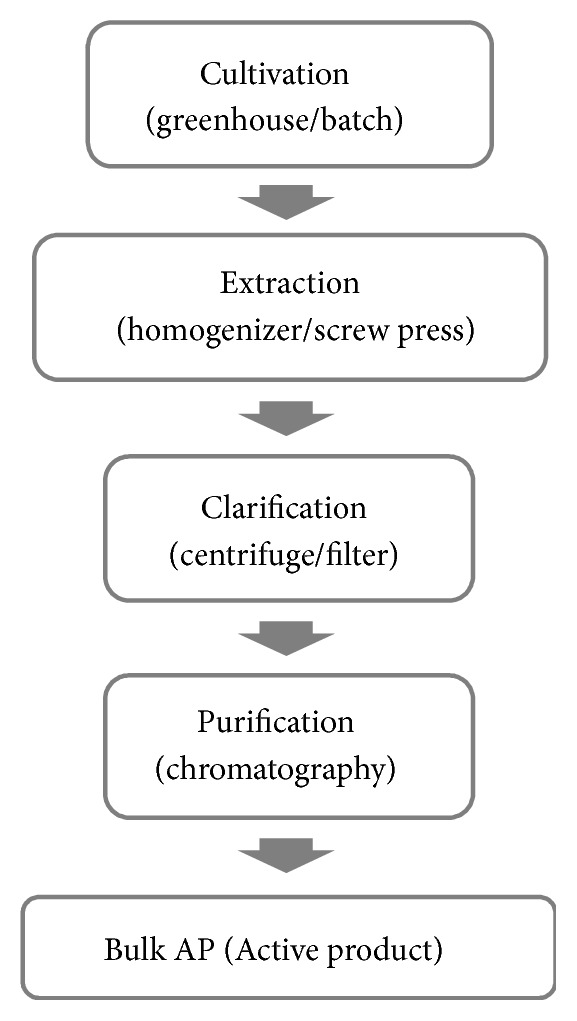
Scheme of the process of large-scale production of recombinant proteins in plant platforms. Based on [39].

When the production of a functional protein is developed at the laboratory stage in the selected expression platform, the scale of production is increased (Figure 1 presents a scheme of the production process on an industrial scale in plant systems). This involves field crops, where each plant serves as a bioreactor and such cultivation can be easily expanded by sowing new individuals or using a vertical farming unit (VFU, fully automated plant-handling facilities complying with current GMP (Good Manufacturing Practice) requirements) [39]. As reported by Byuel et al. (2017) [39], the yields of recombinant protein obtained using VFU reached 2 g kg^−1^ (after optimization). Moreover, they state that the annual demand for 50 t of pure recombinant protein (e.g., monoclonal antibody) can be covered by 72 t y^−1^ of bulk mAb from 3.5–11.0 km^2^ of open fields or 0.4–1.2 km^2^ of VFU area. On the other hand, the same yields of production, but in a CHO-based system (Chinese hamster ovary cells), required a bioreactor volume of 250 000 L. A well-known example of the largest plant-based production system is the one established by Fraunhofer IME to 2G12 (Ab) output. This system allows approximately 900 L of bulk extract to be produced, processing 250 kg of tobacco leaf biomass [39].

At present, some research institutions have developed fermentation strategies (e.g., fed-batch, continuous fermentation) based on a plant system which is easy to scale up [42]. For a plant cell culture-based system, bioreactors for recombinant protein production on a large scale with 1000-25 000 L working volume are usually used [8]. As proven by numerous studies, plant cell systems are successfully used for producing therapeutic proteins on an industrial scale [40, 41]. However, thus far only few examples of therapeutics produced in this way have been commercialized. The main limitation in terms of commercialization is the low protein yield (0.01-10 mg/L). A protein productivity of 10 mg/L was generally considered as the entry level for expanding the commercial process. But recent progress in plant genetic engineering allows the yields of recombinant proteins to be improved to a level of 100 mg/L (e.g., antibodies) or even up to 247 mg/L (e.g., *α*1-antitrypsin) in rice cell culture [42].

A well-known example of a therapeutic protein produced on an industrial scale in carrot cell cultures (ProCellEx™) is the human recombinant *β*-glucocerebrosidase (taliglucerase alfa) enzyme used in the treatment of Gaucher disease [40]. This recombinant protein was approved by the FDA in 2012, whereas the secretory IgA monoclonal antibody, which recognizes the surface antigen I/II of* Streptococcus mutans* (CaroRx™), was approved in Europe as a solution for the prevention of tooth decay [41]. In addition to these therapeutics, many other biopharmaceuticals for both humans and animals are at an advanced stage of market implementation [31].

## 3. Extraction

Proteins can be naturally secreted in eggs, in milk, or, for example, in a mammalian cell culture. Nonetheless, intracellular proteins must be isolated. Unlike bacteria, yeast, or plants, animal cells have no cell wall. In order to extract intracellular proteins, cells first have to be disrupted and then recombinant protein molecules must be released in a soluble fraction. In order to optimize protein stability and extraction efficiency, a suitable detergent should be chosen, specific to a given protein, due to their numerous classes and their different nature. Hence, a detergent factor used for protein extraction not only should be highly efficient but also must diminish protein structural disruption [43]. Appropriate methods should be adopted, e.g., when membrane proteins or proteins from tissues high in lipids, polysaccharides, and other nonprotein components are recovered. Cell lysis can be conducted in many ways, e.g., chemical or mechanical ones, such as osmotic shock lysis, enzyme digestion, sonication, or homogenization [26, 44, 45]. Having extracted the mixture of proteins, the “final” but most troublesome recovery step—purification—can be embarked upon.

## 4. Purification

Although the technology for biotherapeutics production has been changing since the development of the idea of heterologous synthesis, scientists still point to purification as being the most serious obstacle. Proteins used for human therapy, including biopharmaceutics, must not contain superfluous proteins, neither endotoxins nor contaminants. The purification procedure consists in the separation of the target protein, while maintaining its chemical structure and biological activity [46]. Purification processes account for between 45 and 92% of the total costs of manufacturing recombinant proteins [47]. Accordingly, the strategy of purification is crucial in the entire production process. Factors such as large recovery, ease, economic considerations, and reproducibility in laboratories should be taken into account [10]. Usually a chromatography approach is required to meet the obligatory stringency of purity, which sometimes exceeds 99% in the biopharmaceutical industry. The purification process is of great significance, since it plays an unquestionable role in certifying the pureness of the protein drug [12]. The purity, activity, and safety of the finished protein products are ensured by critical aspects including host cell development, cell culture, cell bank establishment, protein synthesis, purification process and subsequent protein analysis, formulation, storage, and handling. The International Council for Harmonisation of Technical Requirements for Pharmaceuticals for Human Use guidelines provide tools for ensuring consistency of this complex process over time. They are source of Process Analytical Technology (PAT) and Quality by Design (QbD) concepts. In general, all biopharmaceuticals across the world (but mostly the ones to be approved by WHO, EU, USA, and Japan) must meet the legal requirements known as Good Clinical Practice, Good Laboratory Practice, Good Manufacturing Practice, and biosafety evaluation. The guidelines cover three main fields of biopharmaceutical production: (1) control of biological sources and raw materials, (2) control of the manufacturing process, and (3) control of the final product. According to the established requirements, biologics produced by different expression systems must be tested for toxicity potential or viral presence (viral clearance processes). The detailed processes of manufacturing and validating biodrugs with regard to host cell type, protein drug type, and acceptable levels of different residual molecules are discussed in an excellent review by Sahoo et al. (2009) [48].

Here, a review of selected common chromatography-based purification methods is presented. Each purification methodology uses a particular feature of proteins. When proteins vary in size, Size Exclusion Chromatography (SEC) is used; when the charge is concerned, Ion-Exchange Chromatography is used; when hydrophobicity is concerned, Hydrophobic Interaction Chromatography (HIC) and Reversed-Phase Chromatography (RPC) are used; and, finally, when ligand specificity is concerned, Affinity Chromatography is used [26].

### 4.1. Affinity Chromatography

Affinity Chromatography is the most popular method of all chromatographic approaches [46]. It allows a specific type of protein to be isolated from a mixture of various unnecessary proteins and contaminants. This method is based on protein affinity to some specific particles. The experimental setup includes a funnel on the top of the column with a beaker. The column is filled with insoluble modified gel beads. A specific chemical group is anchored onto them and the protein of interest showing high affinity towards this group binds to it. Undesirable elements do not attach to these beads and migrate down on account of gravitational pull. Subsequently, they are collected in a beaker. Afterwards, the bound protein of interest must be eluted. This can be accomplished by changing physical parameters (for example, pH or temperature), as well as altering ionic strength, the buffer composition, adding different chelators or competitors [1]. Occasionally, the chemical properties of the target protein are not fully identified. In that case, to gain a strong and selective binding of the target protein to the beads in the column fusion proteins are used. They are created by connecting two genes or more coding originally for single proteins. Additionally, a little fragment of DNA is ligated to the terminus of the gene. It allows its translation in-frame with the protein of interest. This short sequence, known as “tag,” accounts for the robust binding to beads within the column. Tagged proteins are able to attach to an affinity column, while untagged ones are separated by washing out [10].

The polyhistidine- (His-) tag mixed with IMAC (Immobilized Metal Affinity Chromatography) is a commonly used method for recombinant protein purification [49]. The His-tag attached to the N or C terminus of the target protein comprises a sequence of histidine residues (typically six or more). The residues have a high affinity to some metal ions, for example, copper, cobalt, nickel, and zinc [50]. Histidine can bind noncovalently, creating coordination bonds with the metal ions mentioned above, immobilized on a resin. The matrix used in IMAC includes a metal-chelating group, which binds His residues affixed to the recombinant protein. Ni(II)-nitrilotriacetic acid is used most frequently. His-tag binds best to IMAC resin when a near-neutral buffer is used [51]. When the tagged proteins are separated, purification via mild elution may be applied. The binding buffer typically comprises imidazole [51]. Therefore, there is a slight chance of weakening of protein activity or disturbing of its proper folding. In the next stage, it may be necessary to remove the His-tag, which might prove to be costly. On the other hand, as a His-tag sequence is very short, it may not affect the final structure of the protein. However, it could influence the solubility negatively. In that case, large solubility tags are considered, leading to an increase in the total protein solubility. Afterwards, they are normally removed by means of specific proteases, which are removed in a subsequent Affinity Chromatography step. There is an alternative to this protease-based tag elimination system that was designed to lower costs. Specific costly proteases might be replaced with, e.g., self-cleaving intein tags [49].

The method presented here has numerous advantages; generally, it can be used for many recombinant proteins, since His-tag does not possess an electric charge and it is neither immunogenic nor toxic [51]. Moreover, the reagents are commercially available and only a single column is required [49]. On the other hand, the aforementioned heavy metals can leach out from the column, lowering the productivity of the purification.

Immunoaffinity Chromatography is another form of Affinity Chromatography. Here, a specific antibody against the target protein is bound to gel beads. This method is very accurate on account of the antibody-antigen interaction, although two serious drawbacks can be identified. First, to obtain a specific antibody, the purified protein has to be injected into an immunized animal. This involves the application of alternative purification methods first. Moreover, antibodies attach the specific proteins with high affinity, impeding the elution of the proteins from the resin. To obtain it, pH is decreased, although, in some cases, substances like chaotropic agents or urea must be supplied to inactivate the protein [26, 52].

### 4.2. Ion-Exchange Chromatography (IEX)

This kind of chromatography is generally the first step for the separation of protein molecules [47]. Principally, the setup comprises a funnel which is positioned on a column containing gel beads, and thin-layer chromatographic procedures are also used [53]. These gel beads have either a positive or negative charge, which depends on the charge of the target protein. They are usually made from polysaccharides like agarose, cellulose, or dextran linked to side groups, which can exchange ions, for example, diethyl-aminoethyl (DEAE; an anion exchanger) and carboxymethyl (CM; a cation exchanger) [52, 53]. As proteins are amphoteric, they have a tendency to attach to a resin of an ion exchange under the pH of specific conditions. Depending on pH, proteins receive a positive, neutral, or negative charge; hence they can attach to the resin or display no electrical attraction and be washed out from the column. Afterwards, the bound proteins need elution from the ion-exchange resin with a salt solution buffer. The ions present in the salt compete with proteins for room on the resin; therefore the electric bonds of the proteins and the beads break. Subsequently, the proteins, due to the increase in charge, are washed out [52]. This method has proven to be suitable for the separation of proteins of different charges at a specific pH.

### 4.3. Hydrophobic Interaction Chromatography (HIC) and Reverse-Phase Chromatography (RPC)

Proteins comprise amino acids which have hydrophobic and hydrophilic side chains that define their affinity to water. This characteristic plays the main role in protein folding. Hydrophobic Chromatography uses hydrophobic interactions in order to purify medium hydrophobic proteins [52]. Proteins bind to a hydrophobic resin thanks to their hydrophobicity. However, the process requires a high salt concentration in the column. Water molecules surround hydrophobic protein fields, which prevents proteins from binding to hydrophobic ligands on chromatographic media. This can be counteracted by adding ammonium sulfate, for example. In order to obtain bonded proteins, the salt gradient is reduced [47]. The efficiency of the elution process is increased by adding, e.g., alcohols [53].

The reaction scheme of Reverse-Phase Chromatography is similar to Hydrophobic Interaction Chromatography. The key difference concerns chromatography media. In HIC the concentration of the hydrophobic elements bound to the resin is 10-20 *μ*mol/mL, while in RPC it reaches several hundred *μ*mol/mL. This leads to stronger binding in Reverse-Phase Chromatography. It also includes the usage of, e.g., methanol (a solvent less polar than water) during elution. In comparison to Hydrophobic Interaction Chromatography, Reverse-Phase Chromatography poses a higher risk of denaturation of protein molecule, but, at the same time, it is advantageous for qualitative analysis [26, 53].

### 4.4. Size Exclusion Chromatography (SEC)

This method uses the difference in the size of proteins. A mixture of proteins is put into a column filled with pore gel beads, consisting of a buffer and polysaccharide chains. The buffer localizes inside and between the beads. If a large protein is placed on the column, it has no possibility to get into the bead and, as a result, it goes down very quickly. In contrast, a small protein is located inside the gel pores and therefore exits the column later. The Size Exclusion Chromatography separation rate is low. This strategy cannot be used for proteins whose sizes differ marginally [52].

## 5. Conclusions

Due to the fact that 2017 marked 35 years since the first recombinant medicine was approved by FDA, we provided a brief reminder of the available prokaryotic and eukaryotic expression systems and of variations of the “first-to-try,” most established method of purification: chromatography. Proteins and peptides belong to an increasingly important category of biopharmaceuticals. Taking into consideration the fact that within the next 10 years at most around 50% of all the medicines developed will be biopharmaceuticals, it is understandable that both expression systems and associated technologies of protein recovery arouse interest in scientific and business circles [12]. The production of recombinant therapeutics is a complex, multidisciplinary, and expensive process. The time required to implement the initial idea of a therapeutic compound and gain a functional product has been estimated at about 15 years [12]. In spite of this, according to the 15th Annual Report and Survey of Biopharmaceutical Manufacturing Capacity and Production released by BioPlan Associates, Inc. (the organization that has been analyzing the life sciences and biotechnology market for 30 years), the continuously growing world market of biopharmaceuticals has now reached over $250 million (c. $212 million, according to Allied Market Research). Clearly, compared to drugs, biotherapeutics have appeared to be profitable investments. Although, in general, their manufacturing cost is high, biopharmaceuticals are usually developed mostly for diseases that currently lack a good alternative treatment. Consequently, this creates a ready market for them on one hand and supports high prices on the other [54, 55].

Invariably, there is high demand for new and enhanced bioprocessing techniques to make “natural pharming” cheaper and increase its efficiency. The dynamic improvement in biopharmaceutical expression systems demonstrates their potential and tremendous methodological basis. Taking into consideration such promising industrial interest, it is understandable that the development of recombinant proteins is conditioned and eagerly assisted by scientific research circles, where a range of expression vehicles have been created. Which of the systems offers the greatest value is a moot point. Some of them offer lower processing costs, others a low risk of pathogen contamination and further important chemical modifications, while some systems also give rise to fewer ethical objections. Reading scientific papers on particular expression platforms one cannot deny that scientific communities working on microorganisms, plants, or mammalian cells all advertise their respective products, hoping for new strategies and overcoming the existing constraints. Nevertheless, all of them will have to face challenges of downstream processing.

## Figures and Tables

**Table 1:** Comparison of different expression platforms features. Developed based on [1, 7, 8].

**Platform/host**	**Overall costs**	**Production time**	**Scale-up capacity**	**Propagation**	**Product yield**	**Product quality**	**Contamination risk**	**Purification cost**
**Transgenic plants**	very low	medium	very high	easy	high	high	low	high

**Plant cell culture**	medium	medium	medium	easy	high	high	very low	medium

**Plant viruses**	low	low	high	feasible	very high	medium high	very low	high

**Microalgae**	low	high	high	easy	high	high	very low	medium

**Yeast**	medium	medium	high	easy	high	medium	low	medium

**Bacteria**	low	low	high	easy	medium	low	medium (e.g., endotoxins)	high

**Mammalian cell culture**	high	high	very low	hard	medium-high	high	very high (e.g., virus, prions, oncogenic DNA)	high

**Transgenic animals**	high	high	low	feasible	high	high	very high (e.g., virus, prions, oncogenic DNA)	high

**Insect cell culture**	medium	medium	high	feasible	high	medium	very low	medium

**Filamentous fungi**	low	high	high	easy	high	medium	low	low

**Table 2:** Example of biopharmaceutics generated throughout plant-based platform and their current status. Based on [31, 36–38].

Product	Application	Host plant	Status	Company
Recombinant human intrinsic factor	Vitamin B12 deficiency	*Arabidopsis thaliana*	Phase II completed 2006	University in Aarhus, Denmark

Recombinant human lactoferrin	anti-inflammatory conditions in HIV patients	rice (*Oryza sativa*)	Phase I and Phase II, completed 2006, under Phase III	Jason Baker (MMRF), USA Ventria Bioscience, USA

Locteron™, a controlled release interferon	Hepatitis B and Hepatitis C	duckweed (*Lemna minor*)	Phase I and II, completed 2009, under Phase III	Biolex Therapeutics, USA

P2G12 antibody	HIV	tobacco (*Nicotiana tabacum*)	Phase I completed 2011	University of Surrey, Guildford, UK

HIV antibody	HIV	Tobacco (*N. tabacum*)	Phase I	Fraunhofer IME

Insulin (SBS-1000)	diabetes	safflower (*Carthamus tinctorius*)	Phase II, Phase III completed	SemBioSys

Taliglucerase alga	Gaucher disease	carrot (*Daucus carota*) suspension culture (Elelyso™)	Phase III completed 2012, approved by FDA^*∗*^ 2012	Protalix Biotherapeutics Karmiel, Israel

HAI-05	H5N1 vaccine	tobacco (*N. tabacum*)	Phase I, 2011	Center for Molecular Biotechnology, Plymouth, MI, USA

Recombinant interferon (IFN-*α*2b)	antiviral treatment	duckweed (*L. minor*)	Phase II	Biolex Therapeutics, USA

HAI-05	H5N1 vaccine	tobacco (*N. tabacum*)	Phase II, 2012	Medicago Inc., USA & Canada

Human *α*-galactosidase	Fabry disease	Moss (*Physcomitrella patens*)	Phase I	Greenovation Biotech GmbH, Germany

PRX-102	Fabry disease	tobacco suspension culture	Phases I and II, 2014	Protalix Biotherapeutics Karmiel, Israel

Vaccine recombinant protective antigen	Anthrax	tobacco (*N. tabacum)*	Phase I, 2014	Center for Molecular Biotechnology, Plymouth, MI, USA

H5-VLP+GLA-AF vaccine	Influenza A subtype H5N1 infection	tobacco (*N. tabacum)*	Phase I completed, 2014	Infectious Disease Research Institute, Seattle, WA, USA

Vaccine Pfs25 VLP	Malaria	tobacco (*N. tabacum)*	Phase I, 2015	Center for Molecular Biotechnology, Plymouth, MI, USA

ZMApp	Ebola virus	tobacco (*N. tabacum)*	Phases I and II, 2015	National Institute of Allergy and Infectious Diseases (NIAID), Bethesda, MD, USA

CaroRx (Anti-caries)	dental prophylaxis	tobacco (*N. tabacum)*	Discontinued, 2016. 2. 17; Approved as medical device	Planet Biotechnology

Anti-west virus mAb Hu-E16	Anti-west virus	*Nicotiana benthamiana*	Phase II	MacroGenics, NIH

Human Epidermal growth factor	burns treatments	barley seed (*Hordeum vulgare*)	Commercialization	ORF, Sif Cosmetics

Human growth hormone	deficiency treatments	barley seed (*H. vulgare*)	Commercialization	ORF

single-chain Fv (scFv) epitopes	treatment of non-Hodgkin's lymphoma	*N. benthamiana*	Phase I	Large Scale Biology Corp., Vacaville, CA

VEN200 (albumin)	deficiency treatments	rice seed (*O. sativa*)	Phase II	Ventria Bioscience

RhinoRx™	rhinovirus	tobacco (*N. tabacum*)	Phase II	Planet Biotechnology

Newcastle disease virus protein	poultry vaccine	plant cell cultures	USDA approved	Dow AgroSciences

Protein E envelop of Zika virus	Zika virus	*N. benthamiana*	Preclinical trials	Arizona State University, Tempe, AZ, USA
